# Bridging veins: an analysis of surgical anatomy and histology correlated with interhemispheric approaches

**DOI:** 10.3389/fnana.2024.1406252

**Published:** 2024-11-28

**Authors:** Yuanliang Ye, TianCai Lan, Xiangbo Zeng, Jianqing Yang, Ruixiang Wei, Jiale Zhu, Moukun Liu, Xiaowen Zhu

**Affiliations:** ^1^Department of Neurosurgery, Liuzhou People’s Hospital, Liuzhou, Guangxi, China; ^2^Engineering Technological Research Center for Nervous Anatomy and Related Clinical Applications, Liuzhou, Guangxi, China; ^3^Department of General Surgery, Liuzhou People's Hospital, Liuzhou, Guangxi, China; ^4^Department of Intensive Care Unit, Liuzhou People's Hospital, Liuzhou, Guangxi, China

**Keywords:** bridging veins, interhemispheric approaches, superior sagittal sinus, anatomy, endoscopy

## Abstract

Damage to bridging veins could lead to disastrous complications during interhemispheric approaches. We investigated the morphological and histological characteristics of bridging veins. A total of 10 cadaveric heads and 86 patients were analyzed with either anatomic dissection or neuroimaging. The morphological features of the bridging veins and superior sagittal sinus were analyzed by the endoscope. The histology of the junction between the bridging veins and superior sagittal sinus was evaluated under the microscope with staining for H&E, elastic fiber, and Masson’s staining. We found three types of bridging vein configurations in the junction between the bridging vein and superior sagittal sinus: direct connection (type A), vein runs a certain distance below the dural wall tightly (type B), and vein runs a certain distance on the lateral sinus (type C). Valvular-like fibrous cords were present on the opening of type A, trabecular in type B, and arachnoid granules in type C. Loose connective tissue connected the venous wall and dura mater in type A, sinus wall forms the inner wall of the bridging vein in type B, bridging vein accompanied by arachnoid granules in the type C. This classification enables surgeons to predict various bridging vein configurations, followed by safely achieving the optimal dissection during interhemispheric approaches.

## Introduction

**Figure 1:**
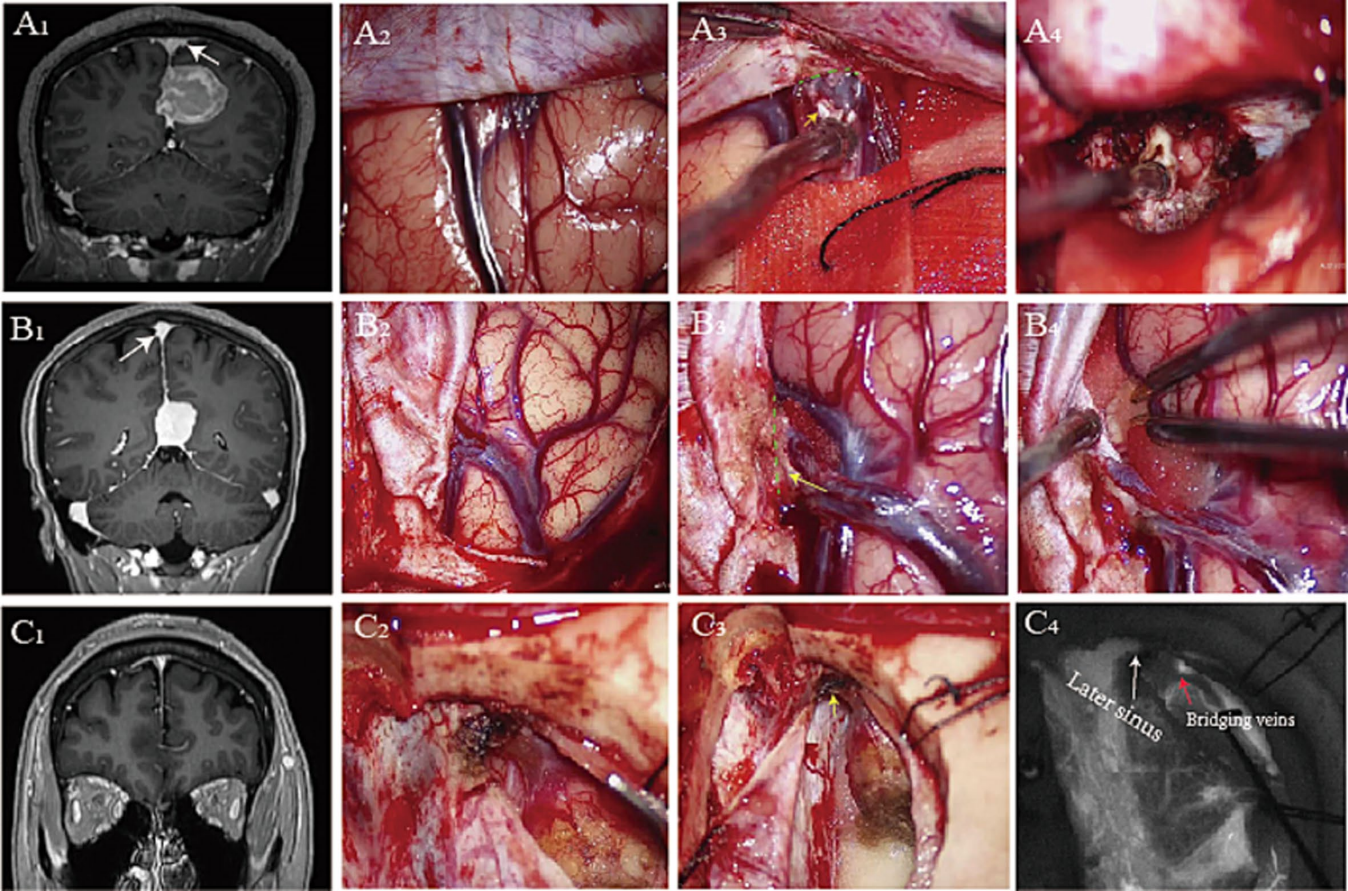
Preoperative postcontrast MR image shows a right falcine meningioma **(A**_**1**_**)**, glioma **(B**_**1**_**)**, and falcotentorial meningioma **(C**_**1**_**)**. Opening the dura mater reveals tight adhesion between the bridging vein and the dura mater **(A**_**2**_**,B**_**2**_**,C**_**2**_**)**. We split the loose connective tissue **(A**_**3**_**)**, partial dura mater **(B**_**3**_**)**, and dura of later sinus **(C**_**3**_**)** to maximum surgical exposure **(A**_**4**_**,B**_**4**_**)** during interhemispheric approaches. Intraoperative fluorescence imaging shows the bridging veins and sinuses **(C**_**4**_**)** (yellow arrowhead: dura mater, green dashed line: surgical interface, white arrowhead: later sinus, red arrowhead: bridging veins).

The surgical corridor between the hemispheres of the brain serves as a gateway to various neurosurgical pathologies. These included distal aneurysms (Kiyofuji et al., 2020), arteriovenous malformations (Agarwal and Barrow, 2019), meningioma (Srinivasan et al., 2021; Casali et al., 2020), cavernous malformation (Elarjani et al., 2021), and pineal tumors (Arzumanov et al., 2024; Bozkurt et al., 2023). An understanding of the venous anatomy was crucial for the execution of interhemispheric approaches (Anetsberger et al., 2022; Ohara et al., 2017). Damage to bridging veins could lead to intraoperative bleeding, postoperative venous cerebral infarction, or hemiplegia during the procedure (Cai et al., 2021; Magill et al., 2016). The Sugita–Kobayashi maneuver was used to release the bridging veins, allowing safe mobilization of the cerebral hemisphere away from the falx, without comprising venous drainage (Mooney and Al-Mefty, 2022; Sugita et al., 1982). During an interhemispheric approaches procedure conducted at the Liuzhou People’s Hospital of Guangxi Medical University, we successfully freed the vein along the interface between the bridging veins and the dura mater (Figure 1). We speculate that partially removing the dura mater or sinus wall could achieve the maximum degree of release in the bridging veins.

Imaging and standard anatomical methods were used to describe the venous configurations and drainage routes (Aldea et al., 2021; Juskys et al., 2022; Tomasi et al., 2020; Famaey et al., 2015; Han et al., 2007). The direction of inflow from the bridging veins into the superior sagittal sinus (SSS) shows high variability (Baltsavias et al., 2015; Brockmann et al., 2011). Using anatomical landmarks, venous configurations and drainage routes were defined as a single vein join, more contiguous veins join, and venous complex joins at the same point (Karatas et al., 2024). In the junction between the bridging veins and SSS, the orientation of the collagen fibers changed at the level of the venous openings, with the luminal diameter becoming narrow (Vignes et al., 2007). Previous studies show the bridging vein and SSS by vascular casting or image analysis, which enhances the subarachnoid segment and subdural segment segments of bridging veins. How the bridging veins run after penetrating the sinus wall was unclear.

In the present study, we track the possible drainage routes of bridging veins and describe the opening of bridging veins in the SSS by endoscopy. Furthermore, we investigated the junction between bridging veins and SSS by H&E staining, Masson’s staining, and elastic fiber staining, aiming to illuminate possible bridging vein separation during interhemispheric approaches.

## Materials and methods

A total of 10 anatomical specimens, obtained from a fresh autopsy, were fixed in 10% formalin solution at the Department of Anatomy of Guangxi Medical University for at least 2 weeks. All the specimens were older than 18 years (Table 1). This study was approved by the ethics committee of the Liuzhou People’s Hospital (IRB approval ID No.KY-E-01-01). The exclusion criteria included cranial cerebral trauma, neurological disease, and diseases affecting the sinuses. The family members signed individual consent giving permission for the use of resected samples for the purpose of research.

**Table 1:** General data of the 10 anatomical specimens and 86 patients.

	M ± SD/%
Anatomical specimens	
Variables	
Mean age (years)	61.2 ± 11.23
Male, no. (%)	6(60%)
Cause of death	
Pulmonary embolism	2
Car accident	4
Acute myocardial infarction	2
Shock	1
Pulmonary infection	1
Patients	
Variables	
Mean age (years)	56.16 ± 16.15
Male, no. (%)	53(61.63%)
Diagnosis, no. (%)	
Suspicion of an intracranial lesion	25(29.06%)
Evaluation of cerebral vein diseases	52(60.46)
Tumor	10(11.63%)
Type of the junction, no. (%)	
Type A	52.17%
Type B	29.59%
Type c	18.24%

### Endoscope assessment

There were six male and four female specimens with a mean age at death of 61.2 ± 11.23 years (range: 43–71 years). Latex was not injected into the vein vessels and sinuses. The scalps were removed, and the cranial vault above the axial plane across the nasion and the inion was removed by surgical power device (Xishan, China). The superior sagittal sinus was flushed with tap water, and blood clots were removed by the insertion of a 4.5-gauge needle. The bridging veins were identified on the surface of the brain. A 4.5-gauge needle was used to slowly inject blue ink into the bridging veins, making them fully visible. During the procedure, we avoided inks entering the subarachnoid space.

With the cadavers in prone, fixed in Mayfield head holder, a high-definition rigid endoscope (Karl Storz, German) measuring 2.7 mm in diameter and optics (0 and 30°) was inserted into the lumen of the sinus from torcula herophili. The endoscopes were attached to a video system and a digital camera, allowing photographic documentation of relevant structures. The opening of the bridging vein in the SSS, dyed with blue pigment, received special attention.

### Dissecting microscope assessment

Bridging veins – SSS complex were carefully removed en bloc by a surgical microscope (OPMI6, Zeiss). The junction between the bridging veins and SSS samples was continuously transversely sectioned with a thickness of 1 cm. The interest observative area included bridging veins, the wall of SSS, arachnoid granules, and dura mater.

### Light microscopy assessment

Following sectioning, the junction between bridging veins and SSS samples was prepared for microscopic assessment. To assess those morphological characteristics, H&E staining was used, and a special staining method was used to detect collagen fibers (Masson’s trichrome) and elastic fibers (Victoria blue). The histological sections were analyzed and documented using a Zeiss Axioskop plus microscope (Carl Zeiss Microscopy) at 40×, 100×, and 400× magnification. Images were acquired and stored using Axio Vision software.

### Enhanced MR venography analysis

The study had 86 patients enrolled, with 53 male and 33 female patients. The mean age at diagnosis was 56.16 ± 16.15 years (range: 30–75 years). MR venography analysis was performed to demonstrate the connection between the bridging vein and the venous sinus dura mater. The indications for MRI-enhanced analysis were suspicion of an intracranial lesion (25 patients) and evaluation of cerebral vein diseases (venous sinus stenosis, venous sinus thrombosis or dural arteriovenous malformations, etc.) (51 patients) or tumor (10 patients) (Table 1). Exclusion criteria were: (1) cerebral vascular diseases involved with the SSS and bridging veins; (2) intracranial tumors involved with the SSS and bridging veins; and (3) the SSS and bridging veins were unclear in the imaging. The bridging veins were tracked by PACS image software and identified the relationship between veins, the dura mater, and venous sinuses. Images were obtained as previously described (Happonen et al., 2023).

### Statistical analysis

All statistical analyses were performed using *SPSS* 23 for Windows (*SPSS* Inc., Chicago, Illinois). The categorical data, including numbers of types of bridging veins and percentages, were summarized using descriptive statistics. The numerical data were expressed as means, SDs, minimums, and maximums.

## Results

### Microscopical observations

**Figure 2:**
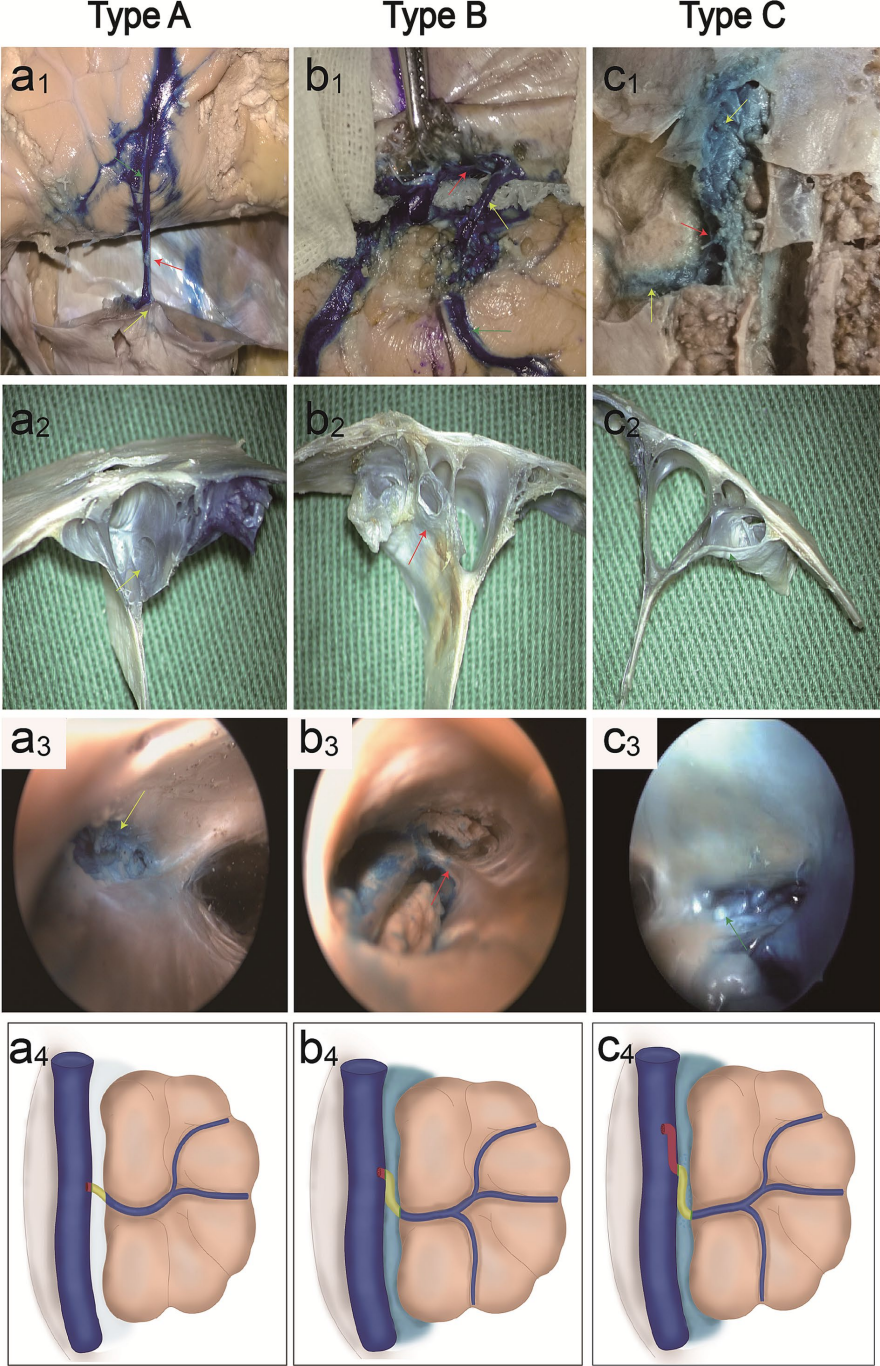
Classification of parasagittal bridging veins. Cadaveric specimen shows vein (red arrowhead) emptying into the SSS, with microscopic anatomy **(a**_**1**_**,a**_**2**_**)** and endoscopic anatomy **(a**_**3**_**)** (yellow arrowheads: opening of the vein). Illustration **(a**_**4**_**)** shows a type A direct connection in the junction between the bridging vein and SSS. Cadaveric specimens show veins run a certain distance below the dural wall, with microscopic anatomy **(b**_**1**_**,b**_**2**_**)** (red arrowhead: vein below dural wall) and endoscopic anatomy **(b**_**3**_**)** (red arrowhead: trabecular-like fibrous cords exist in the opening of veins). Illustration **(b**_**4**_**)** shows type B vein runs a certain distance below the dural wall tightly. Cadaveric specimens show the vein runs a certain distance on the lateral sinus in the SSS, with microscopic anatomy **(c**_**1**_**,c**_**2**_**)** and endoscopic anatomy **(c**_**3**_**)** (arrowheads: arachnoid granules in the opening of the vein). Illustration **(c**_**4**_**)** shows the type C vein runs a certain distance on the lateral sinus.

Bridging veins can be divided into subarachnoid segments, subdural segments, and venous sinus wall segments. The arachnoid granules were located in the subarachnoid space and closely adhered to the dura mater, dividing the bridging vein into subarachnoid and subdural segments. We identified three anatomical types of bridging vein configurations in the junction between the bridging vein and superior sagittal sinus (Table 1): direct connection (type A, Figures 2a_1_,a_2_), vein runs on the dural wall tightly (type B, Figures 2b_1_,b_2_), or vein runs a certain distance on the lateral sinus (type C, Figures 2c_1_,c_2_). One specimen can contain all anatomical types simultaneously.

### Endoscopic observations

Valvular fibrous cords were present on the opening of type A bridging veins (Figure 2a_3_), covering most of the entrance to the superior cerebral vein. The trabecular-like fibrous cords exist in the opening of type B bridging veins (Figure 2b_3_) and divide the opening into different drainage channels. The arachnoid granules were present on the opening of type C bridging veins (Figure 2c_3_). Illustration shows type A direct connection in the junction between the bridging vein and SSS (a_4_), type B vein runs below the dural wall (b_4_), and type C vein runs on the lateral sinus (c_4_).

### Morphological observations

**Figure 3:**
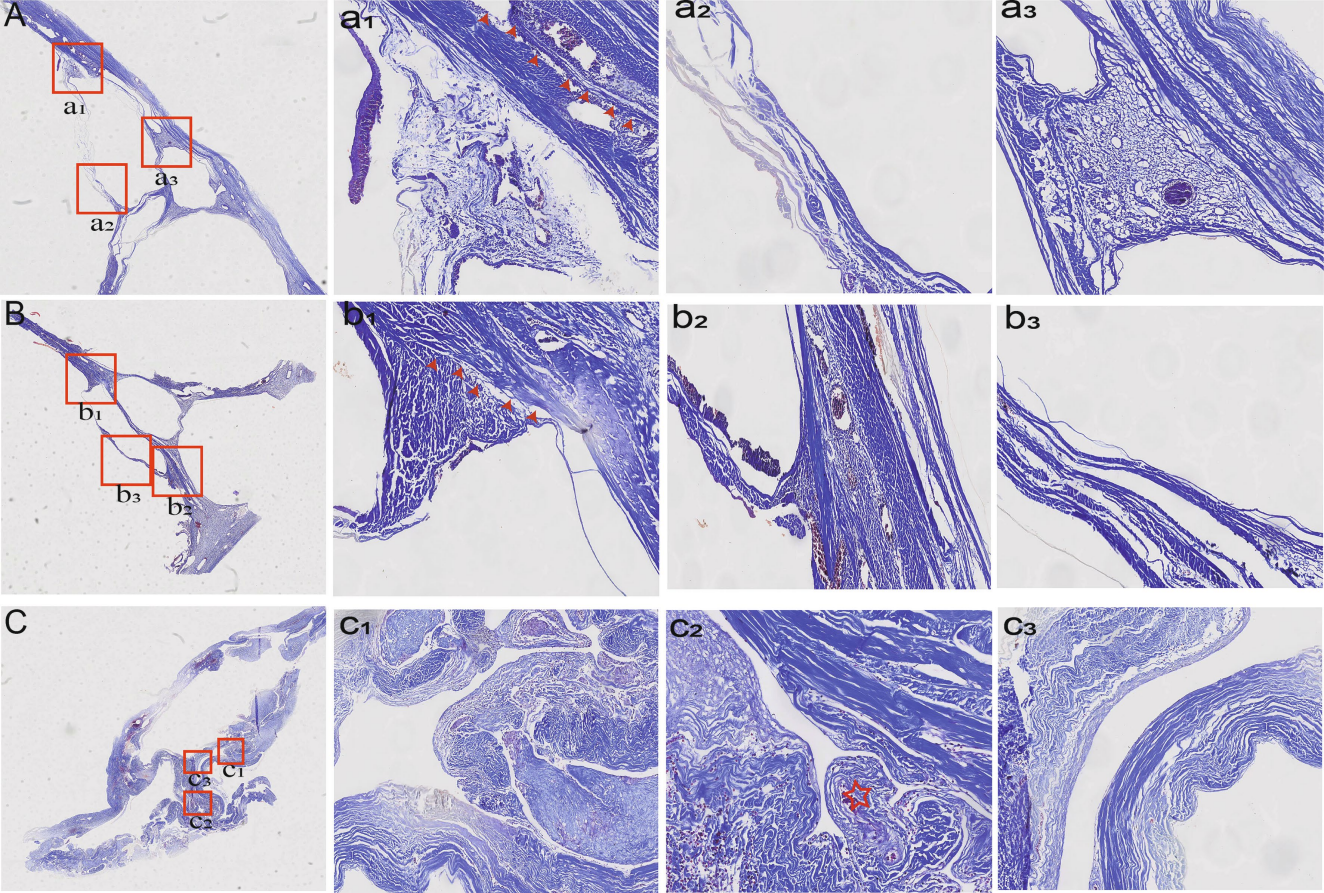
Histological characteristics of different types of bridging veins. In type A, the venous wall was connected by loose **(a1)** (red arrowheads) or dense connective tissue tightly **(a2,a3)**. In type B, collagen fibers were arranged in various layers **(b**_**1**_**,b**_**2**_**)** (red arrowheads). Collagen fibers form the outer walls of the bridging vein **(b3)**. In type C, the vein was found in the lateral sinus **(c**_**1**_**,c**_**3**_**)** and ran inside the arachnoid granules **(c**_**2**_**)** (red asterisk).

The wall of the bridging vein was composed of uniformly arranged wavy collagen fibers. The wall of the venous sinus was composed of dense collagen fibers and arranged in a layered pattern. The junction between the bridging vein and the SSS can be divided into three histological types in Table 2. In type A, loose connective tissue connected the venous wall and dura mater (Figures 3A,a_1_,a_2_), and dense connective tissue connected the venous wall and SSS tightly (Figures 3A,a_3_). In type B, the upper and lower walls of the bridging vein were closely connected to SSS, and collagen fibers were arranged in various layers (Figures 3Bb_1_,b_2_). The sinus wall forms the inner wall of the bridging vein. Collagen fibers form the outer walls of the bridging vein (Figures 3B,b_3_). In type C, the bridging vein was located in the lateral sinus and ran on the inner side of the arachnoid granules, and the venous wall was composed of endothelial cells and a number of collagen fibers (Figures 3C,c_1_–c_3_).

**Table 2:** Different anatomic types of bridging veins merging into venous sinuses.

Type	Anatomic characteristics	Morphological characteristics
A	Direct connection, bridging vein directly merges into the venous sinus	The venous wall was connected to the dura mater by loose connective tissue, while dense connective tissue is tightly connected to the SSS.
B	Bridging vein runs a certain distance on the dural wall tightly, the trabecular exists in the opening of veins.	The bridging vein’s wall had a strong connection to SSS and collagen fibers were arranged in different layers.
C	Bridging veins runs a certain distance on the lateral sinus. The arachnoid granules was present on the opening of veins	Endothelial cells and a significant amount of collagen fibers made up the venous wall.

### Imaging analysis

**Figure 4:**
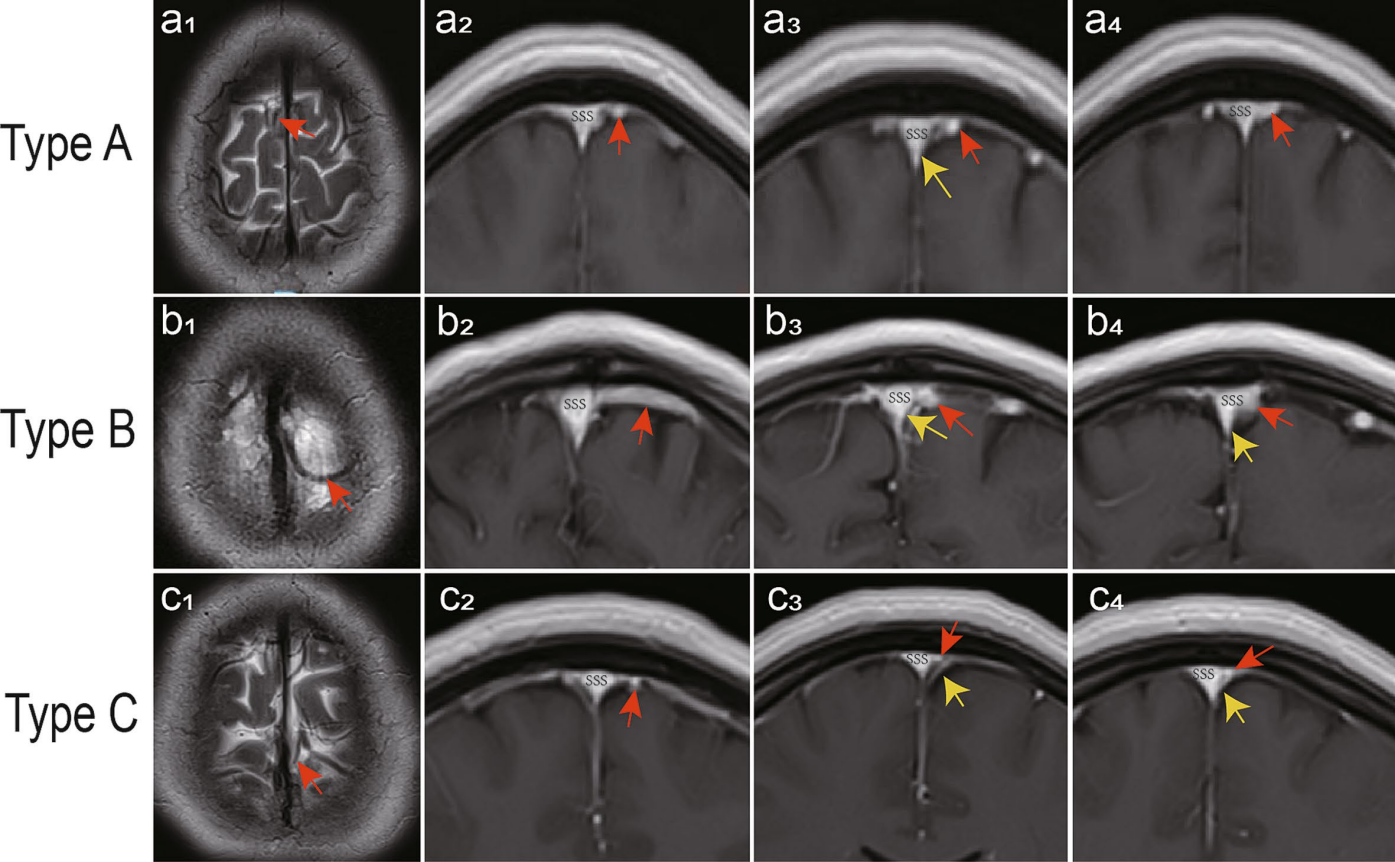
Thin layer imaging analysis in different types of bridging veins (red arrowheads). Type A has veins that run beneath the dura mater (yellow arrowheads) **(a**_**1**_**–a**_**4**_**)**, type B has veins that run on the wall of the venous sinus **(b**_**1**_**–b**_**4**_**)**, and type C has veins that run on the lateral sinus **(c**_**1**_**–c**_**4**_**)**.

**Figure 5:**
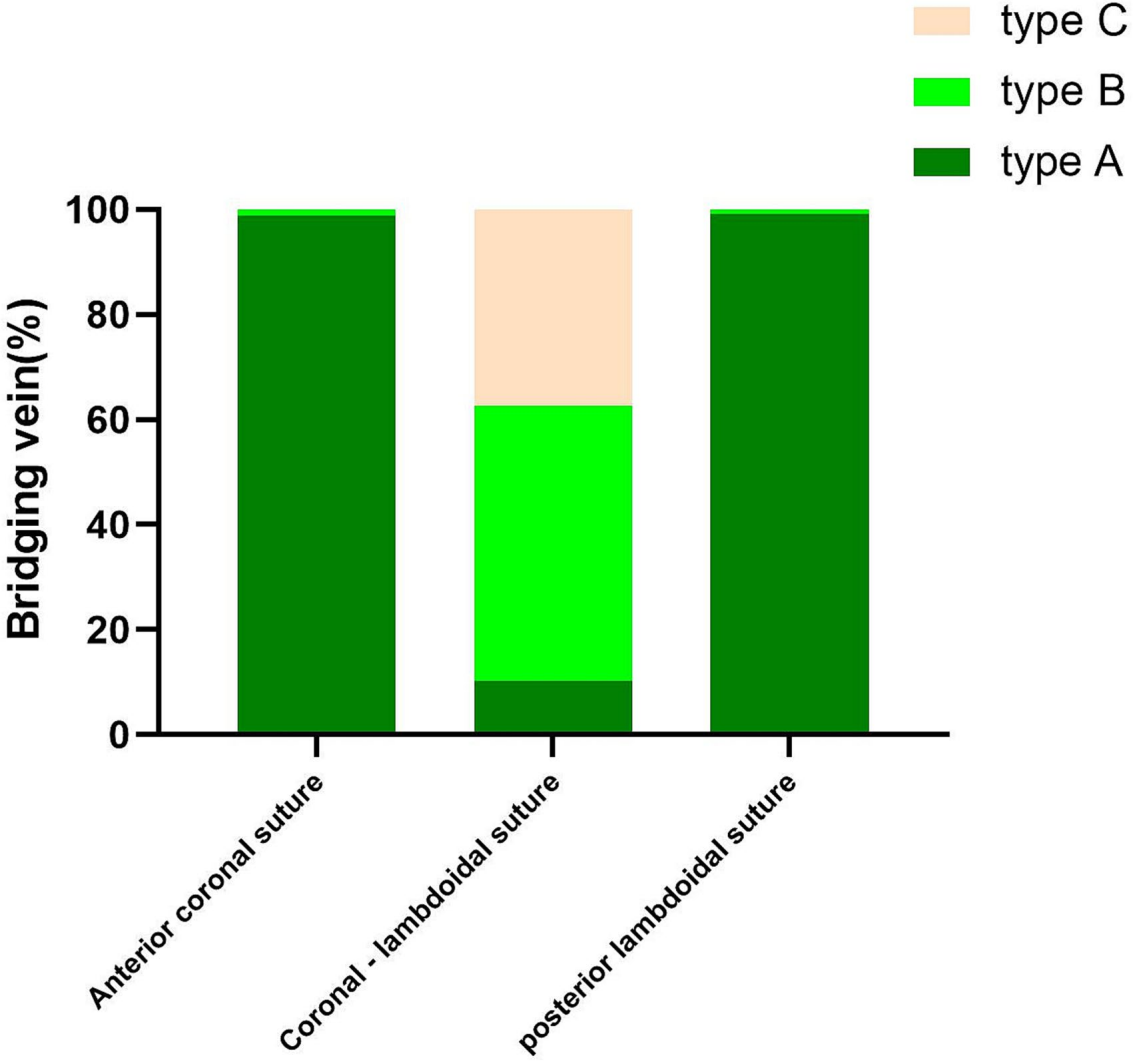
Graphs show comparisons of the number of type A, type B, and type C in different parts of SSS.

With the injection of iodinated contrast material, the three anatomic types at the junction between the bridging vein and SSS were delineated from normal cerebral tissues (Figure 4). Types A, B, and C made up 52.17, 29.59, and 18.24% of all patients, respectively. Moreover, the diameters in the subarachnoid segment, subdural segment, and venous sinus wall (or later sinus) segment were 2.02 ± 1.59 mm, 1.98 ± 1.67 mm, 2.00 ± 1.60 mm in type A, 3.89 ± 2.29 mm, 4.12 ± 2.18 mm, 4.03 ± 2.62 mm in type B, and 1.78 ± 0.89 mm, 1.56 ± 0.64 mm, 1.71 ± 0.89 mm in type C respectively, with statistical significance (*p* = 0.000; Table 3). Type A mainly presented in the area anterior to the bregma or posterior to the lambda, both types B and C appeared on the anatomical area among bregma and lambda in the SSS (Figure 5).

**Table 3:** Comparison of thickness or diameters of bridging vein in different types.

Types	Histological analysis		Imaging analysis		
	Dura wall around BVs (mm)	BVs’s wall (μm)	Subarachnoid (mm)	Subdural (mm)	Sinus wall/later sinus (mm)
A	0.92 ± 0.32	238.24 ± 63.47	2.02 ± 1.50	1.98 ± 1.67	2.00 ± 1.60
B	0.83 ± 0.46	52.61 ± 33.87	3.89 ± 2.29	4.12 ± 2.18	4.03 ± 2.62
C	0.81 ± 0.51	332.7 ± 77.44	1.78 ± 0.89	1.56 ± 0.64	1.71 ± 0.89

## Discussion

This study has demonstrated three types of bridging vein configurations in the junction between the bridging vein and superior sagittal sinus, including direct connection, the vein runs a certain distance on the dural wall tightly and the vein runs a certain distance on the lateral sinus. The arachnoid granules were divided the bridging vein into subarachnoid and subdural segments. Collagen fibers, which are arranged in various layers on the sinus wall, connect the venous wall and SSS tightly. The anatomical and histological characteristics of BVs could result in safe dural incision during interhemispheric approaches.

### Bridging veins drainage routes

Injuries to the parasagittal cerebrovenous structures may lead to devastating complications. Being aware of the inherent bridging veins drainage routes in the region might lower the rate of undesirable outcome. The bridging vein drainage routes were detected by anatomy, cerebral venous computed tomographic angiography (CTA), or digital subtraction angiography (DSA). Some studies propose a systematic classification for the parasagittal venous network, using anatomical landmarks, venous configurations, and drainage routes was defined as a single vein joining in type 1, two or more contiguous veins joining in type 2, and venous complex joins at the same point in type 3 (Karatas et al., 2024). Using CTA with a focus on the direction of the BV entering the SSS, BVs draining into the SSS at the level of the coronary suture typically joined into a lacunar formation rather than proceeding straight through. The second most observed direction of inflow around the coronary suture can be described as a hairpin-shaped flow. Veins draining into the sinus presented a predominantly retrograde inflow direction. An antegrade inflow direction was also seen rostral to the coronary suture (Brockmann et al., 2011). Some studies stated the way BV entered the SSS varied in three dimensions, and thus the BV dura entrance was difficult to precisely localize by DSA, the distribution pattern of the dural entrance of the BVs into the SSS was relatively constant, and a no tributary segment of the SSS was centered at the coronal suture and was identifiable by DSA, and all the BVs entered the SSS at an angle opposite to the direction of blood flow segment was identifiable by DSA (Han et al., 2007). Our study proposed the classification based on the histological and imaging relationship between the dura mater and the bridging vein. The classification clarifies the surgical interface, making it more practical.

Previous studies show the bridging vein and SSS by vascular casting with acrylonitrile butadiene styrene resin in acetone or image analysis. Our study tracked the bridging vein drainage routes by injecting blue ink into the bridging vein and endoscopy, displaying the venous sinus wall segment of BV. We found types of bridging vein configurations including direct connection (type A), vein running a certain distance on the dural wall tightly (type B), and vein runs on the lateral sinus (type C). Those anatomic types were confirmed through enhanced magnetic resonance imaging in patients.

### Junction between bridging veins and SSS

Many studies focused on both bridging veins and SSS by histological analysis. The general structure of a venous wall consists of three layers. The strong tunica adventitia covers the outside of the vein. The second layer is called the tunica media, consisting of smooth muscle cells and elastin fibers. The inner layer or tunica intima is composed of multi-layered smooth endothelium covered by elastin tissue. Microscopy of sinus wall transverse sections indicated the existence of a single layer or a multiple-layered dura sinus wall (Ye et al., 2022; Amato et al., 2016). The wall thickness of the dural sinus was variable in the posterior cranial fossa (Balik et al., 2019). In the junction between the bridging veins and SSS, the venous endothelium stretched beyond the sinus endothelium, and the orientation of the collagen fibers changed at the level of the venous openings, with the luminal diameter becoming narrow and oval-shaped (Vignes et al., 2007; Ye et al., 2022). In our study, we found loose connective tissue connected the venous wall and SSS, sinus wall forms the inner wall of the bridging vein in the junction between the bridging veins and SSS. Arachnoid granules and chordae were presented on the opening of bridging veins.

### Embryogenesis of bridging vein drainage

In embryonic development, the superficial venous system of the brain starts from the primitive cephalic veins, which are initially located on the inner side of the cranial nerves, gradually extending outward to connect the main vein (Raybaud, 2010; Mortazavi et al., 2013). In the fifth week, the superficial venous system was obstructed by the ear vesicles, resulting in the formation of dorsal collateral venous plexus. Some of those collateral venous plexus penetrated the dura mater, while others penetrated the pia mater (Pearl et al., 2011). The dorsal collateral venous plexus is divided into three groups for drainage into the primitive cephalic vein: the anterior group receives drainage from the forebrain and midbrain veins, the middle group drainage of the posterior brain, with the drainage trunk located between the trigeminal ganglia and ear vesicles, and the posterior group drains the terminal brain. Based on the development of brain tissue, skull, and dural sinus, many variations, including bridging vein configurations, drainage routes, and the dural entrance of bridging veins, appeared in the subsequent.

### Clinical significance

**Figure 6:**
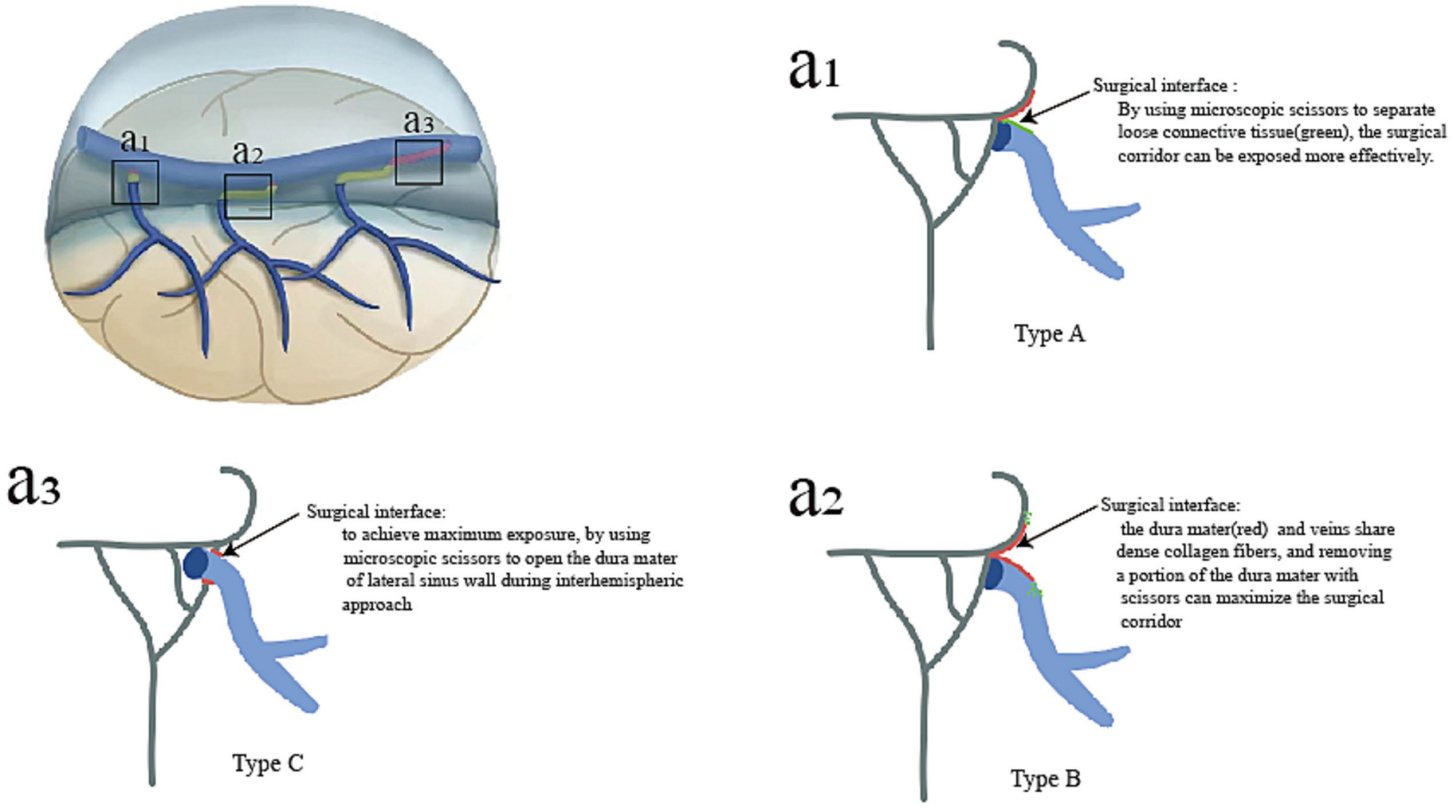
For different types of bridging veins, the various surgical techniques used to separate bridging veins during interhemispheric approaches are summarized. In type A, the maximum surgical exposure was reached when the arachnoid membrane was separated from the surface of the vein **(a**_**1**_**)**. In type B, the maximum surgical space was achieved by dissecting the sinus walls **(a**_**2**_**)**. In type C, a portion of the venous sinus wall around veins could be removed **(a**_**3**_**)**.

Opening the roof of the interhemispheric microsurgical corridor to access various neuro-oncological or neurovascular lesions can be demanding because of the multiple bridging veins that drain into the sinus with their highly variable, location-specific anatomy. Opening the dura mater with maximum safety was beneficial for increasing the surgeon’s working space and degree of brain movement, thus, avoiding inadvertent avulsions, bleeding, and venous thrombosis. Based on the anatomical and histological characteristics of BVs and SSS, we summarize the different dural incisions during interhemispheric approaches (Figure 6). In type A, both loose connective tissues and arachnoid membrane are located in the space between the dura mater and veins. By using microscopic scissors to separate loose connective tissue, the surgical corridor can be exposed more effectively. In type B, the dura mater and veins share dense collagen fibers, and removing a portion of the dura mater with microsurgical scissors can maximize the surgical corridor. In type C, due to the veins running within the lateral sinus, achieve maximum exposure, by using microscopic scissors to open the dura mater of the lateral sinus wall during interhemispheric approach. Preventing intraoperative venous bleeding could be achieved by maintaining tension while pulling the dura mater and following the correct surgical interface.

### Limitations

We recognize that our study has several limitations. First, cadaveric heads vascular replica did not perfectly reflect the flexibility of intracranial vessels. Second, it was difficult to identify types of bridging veins drainage during surgery identify types of bridging vein drainage during surgery; however, intraoperative fluorescence imaging may solve this problem. Third, more clinical practice was needed to prove the interface between bridging veins and the sinus wall.

## Conclusion

This study uses anatomical and histological methods to reveal the different types of bridging veins. Meanwhile, the morphological structure of the junction between bridging veins and SSS was described. Based on the anatomic characteristics of bridging vein configurations, we speculated that the different surgical techniques used to separate bridging veins during interhemispheric approaches guide the surgeon to extend their working space to avoid venous injury.

## Data Availability

The original contributions presented in the study are included in the article/supplementary material, further inquiries can be directed to the corresponding author.
